# Monitoring Mycoparasitism of *Clonostachys rosea* against *Botrytis cinerea* Using GFP

**DOI:** 10.3390/jof8060567

**Published:** 2022-05-26

**Authors:** Rakibul Hasan, Binna Lv, Md. Jamal Uddin, Yingying Chen, Lele Fan, Zhanbin Sun, Manhong Sun, Shidong Li

**Affiliations:** 1 Institute of Plant Protection, Chinese Academy of Agricultural Sciences, Beijing 100193, China; rakib.ppath@sau.ac.bd (R.H.); lvbinna03@163.com (B.L.); jamal_ag@yahoo.com (M.J.U.); cyy150509107270612@163.com (Y.C.); caasfanlele@163.com (L.F.); twins5616@126.com (Z.S.); 2 Department of Plant Pathology and Seed Science, Sylhet Agricultural University, Sylhet 3100, Bangladesh

**Keywords:** *Clonostachys rosea*, GFP, fungal interaction, mycoparasitism, *Botrytis cinerea*, biocontrol, pathogenic fungi

## Abstract

*Clonostachys rosea* is an important mycoparasite, with great potential for controlling numerous plant fungal diseases. Understanding the mechanisms and modes of action will assist the development and application of this biocontrol fungus. In this study, the highly efficient *C. rosea* 67-1 strain was marked with the green fluorescent protein (GFP), and the transformant possessed the same biological characteristics as the wild-type strain. Fungal interactions with *Botrytis cinerea* during co-culture and encounter on tomato leaves were assessed by fluorescence confocal and electron microscopy. The results indicated that once the two fungi met, the hyphae of *C. rosea* grew alongside those of *B. cinerea*, then attached tightly to the host and developed special structures, via which the biocontrol fungus penetrated the host and absorbed nutrients, eventually disintegrating the cells of the pathogen. Mycoparasitism to *B. cinerea* was also observed on tomato leaves, suggesting that *C. rosea* can colonize on plants and act following the invasion of the pathogenic fungus.

## 1. Introduction

Mycoparasitism is a common interaction in nature, in which a living fungus is parasitized by another fungus [1,2]. During the interaction, a mycoparasite perceives the presence of a potential host, grows towards and attaches to it, then often coils around it and forms infection structures that assist host penetration [3]. During the mycoparasitic process, various cell-wall-degrading enzymes (CWDEs) and antifungal metabolites are secreted to digest and kill host cells and uptake nutrients [4,5]. Mycoparasitism is an essential mechanism in the biocontrol fungi to fight against plant fungal diseases. Several mycoparasites have been studied for decades, among which *Trichoderma* spp. [6,7,8], *Gliocladium* spp. [9,10,11] and *Coniothyrium minitans* [12,13] are the most widely used biocontrol agents in the greenhouse and the field. However, unpredictable efficacy in the field sometimes hinders their application [14]. Understanding the interactions and the modes of action of the mycoparasites will promote their control effects to plant diseases and facilitate their application in the field.

To explore mycoparasitism, interactions between different fungal species are observed by microscopy [15,16,17]. Furthermore, molecular techniques involving constitutive expression of fluorescent proteins can be employed to visualize the associations of mycoparasites and hosts, and their colonization in soil and other non-sterile environments. [18]. In recent decades, the gene-encoding green fluorescent protein (GFP), originally isolated from the jellyfish *Aequorea victoria* [19], has been widely used as an effective molecular marker in many prokaryotes and eukaryotes without damaging cell activities. For example, GFP was expressed in *Trichoderma* species, which elucidated their interactions with *Pythium ultimum*, invasion of the hyphae and sclerotia of *Rhizoctonia solani* [4,20], and penetration of the plant parasitic nematode *Globodera pallida* [21]. Using a GFP marker, Németh [22] first visualized *Ampelomyces quisqualis*, a mycoparasite of powdery mildew, and deciphered its lifestyle in the environment, before and after acting as a mycoparasite.

*Clonostachys rosea* (syn. *Gliocladium roseum*) is an attractive mycoparasite capable of invading various phytopathogenic fungi, including *R. solani*, *Sclerotinia sclerotiorum*, *Botrytis cinerea* and *Fusarium* spp. [23,24,25]. Up to now, *C. rosea* preparations have been used in vegetables, ornamentals, and herbs to resist fungal diseases, and good results have been achieved in the greenhouse and the field [26,27,28]. Multiple biocontrol mechanisms have been reported for *C. rosea*, including antagonism, mycoparasitism, competition and induction of plant resistance [25,29], among which mycoparasitism plays an essential role during the biocontrol process. In previous studies, *C. rosea* isolates were successfully labelled with GFP, and their association with barley roots [30] and infection of the saprophytic nematode *Panagrellus redivivus* [31] were investigated. However, mycoparasitic interactions between *C. rosea* and pathogenic fungi remain unclear.

*C. rosea* 67-1 strain (namely *C. chloroleuca* 67-1) [32] is a highly efficient biocontrol fungus, targeting many plant pathogenic fungi [33,34]. In this work, a GFP-tagged 67-1 transformant was constructed, and its mycoparasitic activity against *B. cinerea* was investigated under in-vitro conditions and on tomato leaves. The research provides new insight into the mechanisms of mycoparasitism of *C. rosea* and will facilitate the development of the biocontrol fungus against plant fungal diseases.

## 2. Materials and Methods

### 2.1. Fungal Isolates and Plasmids

*C. rosea* 67-1 strain (ACCC 39160) was isolated from the soil of a vegetable yard in Hainan Province, China, by the sclerotia baiting method [35]. *B. cinerea* TC-B1 and *S. sclerotium* Ss-H were isolated from tomato plants with grey mould and *Sclerotinia*-infected soybean stems, respectively [36]. All isolates were cultured on potato dextrose agar (PDA) and preserved in 20% glycerol at −80 °C in the Biocontrol of Soil-borne Diseases Lab, Institute of Plant Protection (IPP), Chinese Academy of Agricultural Sciences (CAAS).

The recombinant plasmid pSC003 containing the GFP gene, glyceraldehyde-3-phosphate (gpd) promoter and trpC terminator was provided by Hao Zhang of the IPP and used to construct the GFP-labelled strain.

### 2.2. Plant Cultivars

The soybean cultivar Zigongdongdou No. 6 was provided by the Institute of Crop Sciences, CAAS, and the tomato cultivar Jiafen No. 201 was obtained from the Institute of Vegetables and Flowers, CAAS.

### 2.3. Construction of GFP-Labelled Strains of C. rosea

#### 2.3.1. Protoplast Preparation and GFP Transformation

The 67-1 strain was cultured on PDA for 10 days. The spores were eluted, transferred into potato dextrose broth (PDB) and incubated in a fermentation shaker at 180 rpm at 28 °C overnight. The tine hyphae were treated with 40 mg/mL snail enzyme (XJK Biotech, Beijing, China) and incubated at 28 °C and 100 rpm for 3 h. The released protoplasts were collected with a sterilized double-layer microfiber cloth, suspended in STC buffer (200 g sucrose, 50 mL of 1 M Tris-HCl pH 8.0, 5.55 g CaCl_2_ in 1 L distilled water), and stored on ice [37].

PEG-CaCl_2_-mediated transformation of GFP was conducted [38]. The pSC003 plasmid was propagated in *Escherichia coli* DH5α (Transgen Biotech, Beijing, China) in Luria-Bertani (LB) medium with 100 μg/mL ampicillin, and the plasmid DNA was extracted using a TIANprep Rapid Mini Plasmid Kit (Transgen Biotech, Beijing, China). The protoplast suspension was mixed with the linearized plasmid and incubated on ice for 20 min, then 1.25 mL of 40% PTC solution (400 g polyethylene glycol 4000, 10 mL of 1 M Tris-HCl pH 8.0, 20 mg CaCl_2_ in 1 L distilled water) was added. After 20 min, the mixture was transferred into TB3 medium (3 g yeast extract, 3 g casein acid hydrolysate, 200 g sucrose in 1 L distilled water) and cultured at 28 °C and 100 rpm for 16 h.

#### 2.3.2. Verification of GFP Transformants

The suspension of *C. rosea* was mixed with 10 mL TB3 medium containing 200 μg/mL G418 and 0.7% agarose and incubated at 26 °C for 2−3 days. The emerged colonies were picked and transferred onto the resistant PDA plates. After four generations, stable transformants were checked under an LSM 980 fluorescence confocal microscope (Carl Zeiss Microscopy GmbH, Jena, Germany). DNA was extracted from the transformants using a Bio Spin Fungus Genomic DNA Extraction Kit (Takara, Dalian, China). The primers GFPF 5′-GTGACCACCTTCACCTACGG-3′ and GFPR 5′-TGTACAGCTCGTCCATGCC-3′ were designed using Primer3Plus Software (Premier, Boston, MA, USA), and PCR amplification was performed in a 25 µL reaction system containing 23 µL PCR mix, 0.5 µL of each primer, and 1 µL DNA template. Thermal cycling was performed with an initial denaturation step at 98 °C for 3 min, followed by 35 cycles at 98 °C for 10 s, 56 °C for 10 s and 72 °C for 1 min, and a final elongation at 72 °C for 8 min.

#### 2.3.3. Growth and Sporulation of the Transformants

The transformants exhibiting strong fluorescence were selected for bioassay. The isolates were incubated on PDA for 10 days, and the spore suspension of 1 × 10^7^ spores/mL was prepared. Five microliters of the suspension were inoculated on a 5 mm sterilized filter paper on the centre of a 9 cm PDA plate. After incubation at 26 °C for 7 days, the diameter of each colony was measured. The spores were eluted with 5 mL of 0.05% Tween-80, and the spore yield was counted under a BX41 microscope (Olympus, Tokyo, Japan). Three replicates were conducted for each isolate.

#### 2.3.4. Efficacy of GFP-Tagged Isolates on Soybean Sclerotinia Stem Rot

The control efficacies of the wild-type strain and GFP-tagged transformants against soybean Sclerotinia stem rot were tested in the greenhouse. Soil collected from a vegetable yard was mixed with 20% nursery substrate and filled in plastic pots (dia. 11 cm), in which three soybean seeds were sown. When six compound leaves had grown, 100 mL of *C. rosea* suspension (1 × 10^7^ spores/mL) was sprayed onto them. After drying, an equal volume of *S. sclerotiorum* fermentation liquor was smeared with a brush. Seedlings treated with sterilized water and *S. sclerotiorum* broth served as the control. Twelve pots were planted for each isolate, and all pots were arranged randomly and maintained at 26−28 °C in the greenhouse. After 7 days, disease indices on all unfolded leaves were determined using a 0−4 grade scale according to lesions on the leaves [36]. Three replicates were conducted for each treatment.

### 2.4. Mycoparasitism of C. rosea to B. cinerea under In Vitro Conditions

A transformant with the same biological characteristics as the 67-1 strain was selected, named G67-1, and its mycoparasitic actions to *B. cinerea* were investigated on slides with and without nutrients. The G67-1 and TC-B1 strains were incubated on PDA at 26 °C for 7 days, and the agar strips (2 × 0.5 cm) of both isolates were cut from the edges of the fungal colonies with a sterilized scalpel. A sterilized glass slide (8 × 3 cm) was placed on the centre of a PDA plate, and a strip of *C. rosea* agar was laid on one side adjacent to the slide. The fungus was cultured at 26 °C for 2 days, and a strip of *S. sclerotiorum* was laid on the opposite side. In another run, 5 mL of melted PDA was poured into a 9 cm Petri dish, and a sterilized slide was gently submerged into the medium. After solidification, a thin layer of nutrients formed on the surface of the glass, and the two fungi were inoculated successively on both sides. The plates were sealed with Parafilm, and the fungi were co-cultured at 26 °C in an incubator. After 3−4 days, when the two fungi were observed to contact each other (0 h), the slides were taken out, and a droplet of sterile distilled water was added. Interactions between the fungi on the slides were observed under a fluorescence confocal microscope (Carl Zeiss Microscopy GmbH, Göttingen, Germany). A set of fluorescent filters were employed, including a dichroic mirror (495 nm), an excitation filter (450−490 nm) and a barrier filter (500−550 nm). Photographs were captured by an AxioCam ICc5 camera (Carl Zeiss Microscopy, LLC, Thornwood, NY, USA) and processed using Zen 2011 Software (Carl Zeiss Pte. Ltd., Singapore, Singapore). Minor photo editing was performed using Adobe Photoshop (Adobe Systems Incorporated, San Jose, CA, USA) without any changes to the content. The mycoparasitic process was monitored every 24 h, and 10 slides were assessed until the degradation of the host was observed.

### 2.5. Histological Observation by Scanning Electron Microscopy (SEM)

The confrontation of *C. rosea* and *B. cinerea* was also monitored by SEM. The agar strips (2 × 0.5 cm) were cut from the edges of *C. rosea* and *B. cinerea* colonies and placed 3 cm from each other on the surface of 5 mL PDA in a 9 cm Petri dish. The plates were sealed with Parafilm, and the fungi were co-cultured at 26 °C for 6 days until G67-1 strain overgrew *B. cinerea* colonies. The conjoint regions containing both fungi were cut into ~5 mm blocks, fixed in 3% glutaraldehyde at 25 °C in the dark for 48 h, and stored at 4 °C in a refrigerator before observation. Three replicates were included for each sample. The specimens were gently dried using a Leica EM CPD030 instrument (Leica Microsystems, Australia) and coated with gold powder. Interactions between the mycoparasite and its host were detected under a Hitachi SU8010 scanning electron microscope (Hitachi High-Technologies Co., Tokyo, Japan) with an accelerating voltage of 10 kV.

### 2.6. Interaction of C. rosea and B. cinerea in Tomato Plants

Infection of *B. cinerea* by *C. rosea* isolates was investigated on tomato leaves in the greenhouse. Soil collected from an experimental field of the Institute of Plant Protection, CAAS, Langfang, China, was mixed thoroughly with nursery substrate (4:1, *v*/*v*) and filled in 11 cm plastic pots. The tomato seeds were surface sterilized with 2.5% NaClO for 10 min, rinsed three times with distilled water, and sown in a nursery tray (54 × 28 cm, 10 × 5 holes). After 20 days, the seedlings were transplanted into the pots, two seedlings per pot. After growing for 7 days, the tomato leaves were inoculated with 100 mL of GFP-labelled *C. rosea* suspension at a concentration of 1 × 10^7^ spores/mL and *B. cinerea* fermentation liquor successively. Relative humidity was maintained at 90%, and the temperature in the greenhouse was maintained at 26−28 °C.

Five days after inoculation, the leaves were picked and cut into 1 cm discs with a puncher. Mycoparasitism of *C. rosea* against *B. cinerea* on the surface of the leaflets was observed under a confocal scanning microscope (Carl Zeiss Microscopy GmbH, Jena, Germany). Simultaneously, the leaflets were surface sterilized with 2.5% NaClO for 3 min, rinsed with sterile distilled water, and transferred onto 1/4 PDA plates to examine infection of *B. cinerea* and colonization of *C. rosea* in tomato leaves. After 7 days, the fungal colonies were morphologically identified.

### 2.7. Statistical Analysis

Analysis of variance (ANOVA) was assessed using the Package agricolae v4.1.1 (R foundation for statistical computing, Vienna, Austria). The growth and sporulation of the fungal strains were compared by *t*-test, and their biocontrol activities were evaluated by Fisher’s least significant difference (LSD) test at the 5% significance level.

## 3. Results

### 3.1. Stability of GFP-Labelled Strains

**Figure 1:**
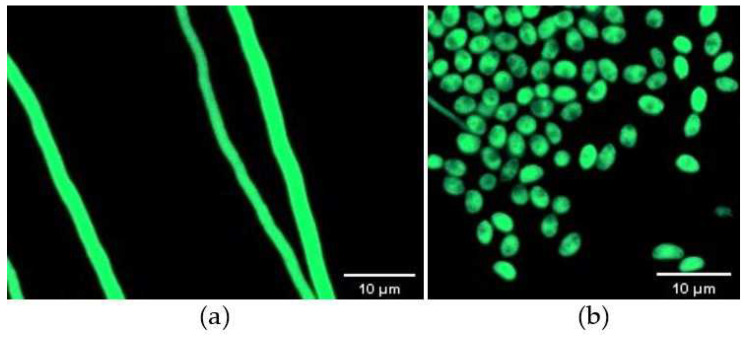
Confocal microscopy images of GFP-labelled *C. rosea* 67-1 transformants observed under phase-contrast light. (**a**) Vegetative hyphae. (**b**) Conidia.

**Figure 2:**
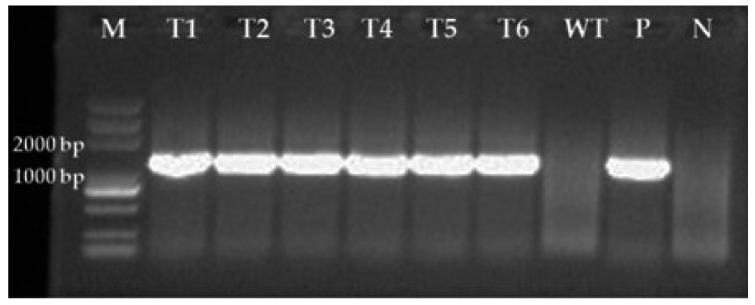
PCR verification of GFP-labelled C. rosea transformants using primers GFPF and GFPR. M, DNA markers; T1−T6, Transformants; WT, Wild-type strain; P, Plasmid; N, Negative control.

**Figure 3:**
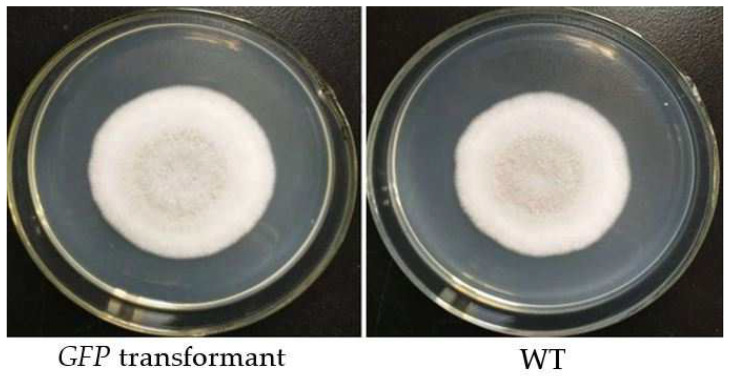
Colony morphology of GFP–labelled *C. rosea* 67-1 transformant on PDA plate after 7 days. WT, Wild-type strain.

A total of 206 mutants grew on TB3 medium containing G418, and all 20 isolates tested emitted green fluorescence under a fluorescence microscope, among which 6 displayed strong excitation (Figure 1a,b). PCR verification showed that all tested mutants yielded a single band of approximately 1297 bp, indicating GFP was successfully inserted into the genome of the *C. rosea* 67-1 strain (Figure 2). The morphology of the transformants exhibiting the strongest fluorescence was quite similar to that of the wild-type strain (Figure 3), and their growth diameters and spore yields were 4.5 cm and 5.4 × 10^7^ spores/plate after 7 days, compared with 4.7 cm and 5.7 × 10^7^ spores/plate for the wild-type strain, which showed no statistical difference (*p* < 0.05). In the pot experiment, the G67-1 and 67-1 strains also showed consistent control effects against soybean Sclerotinia stem rot (*p* < 0.05, Table 1). The above results indicate that the GFP-labelled G67-1 strain can function as the 67-1 strain.

### 3.2. Interactions between C. rosea and B. cinerea under In Vitro Conditions

**Figure 4:**
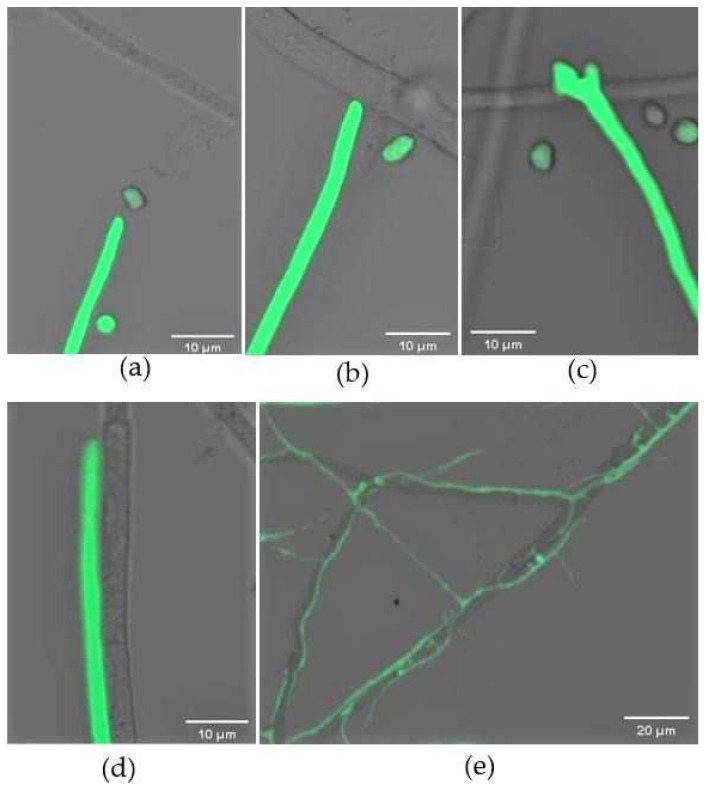
Interaction between *C. rosea* G67-1 and *B. cinerea* TC-B1 strains confrontation cultured on slides within 0−48 h. (**a**) Hyphal extension of G67-1 towards the mycelia of TC-B1. (**b**) Attachment of G67-1 hyphae to those of its host. (**c**) Papilla formed at the tip of G67-1 hyphae. (**d**) G67-1 hyphae growing alongside its host. (**e**) Hyphal branches of G67-1 growing on and around its host.

When co-cultured on the opposite sides of the plate, the hyphae of *C. rosea* and *B. cinerea* extended across the slide. Under a microscope, we could see that *C. rosea* grew towards its host over time (Figure 4a). After ~24 h, the hyphae of *C. rosea* attached to the host (Figure 4b), during which a swollen papilla-like structure formed on the tip of the hyphae (Figure 4c). The mycoparasite hyphae were tight against those of its host and grew alongside them (Figure 4d). On glass slides covering PDA medium, both fungi grew faster than those on slides without nutrients. However, an extra 12 h was needed to produce infection structures for mycoparasitizing the host, implying *C. rosea* might more easily utilize available nutrients. During the mycoparasitic process, the biocontrol fungus branched profusely, and it was clearly seen that *C. rosea* branches grew on and around the hyphae of its host (Figure 4e).

**Figure 5:**
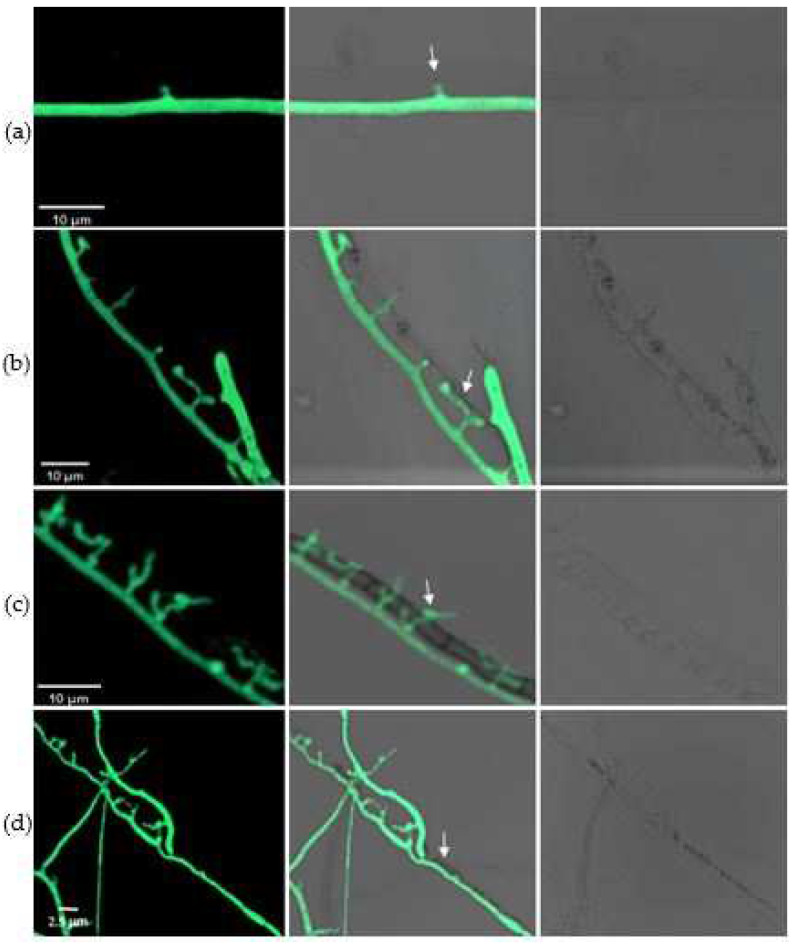
Infection structures formed in *C. rosea* G67-1 strain against *B. cinerea*. (**a**) Hook-like structures of G67-1 formed at 72 h after encountering *B. cinerea*. (**b**) Production of appressoria and penetration into host hyphae. (**c**) Appressorium branches produced inside host cells. (**d**) Disintegration of *B. cinerea* after 6 days. Mycoparasitic interaction between the two isolates was observed using a fluorescence microscope under phase-contrast, differential interference contrast (DIC), and normal fields.

During the mycoparasitic interaction, some specific structures formed that were essential for penetrating the host. At 72 h after encounter, a unique hook-like structure was evident in the mycelia of *C. rosea* (Figure 5a). Appressoria then developed rapidly, and the invasion of the pathogen was achieved via these structures (Figure 5b). Once penetrating into the cells, the mycoparasite branched inside the host and used the pathogen as a nutrient source (Figure 5c). After 6 days, the hyphae of the pathogen gradually became damaged, and were eventually completely destroyed due to infection with the biocontrol fungus (Figure 5d). The penetration structures also developed in *C. rosea* on the nutrient-supplied slips, but this was dependent on the richness of the medium.

**Figure 6:**
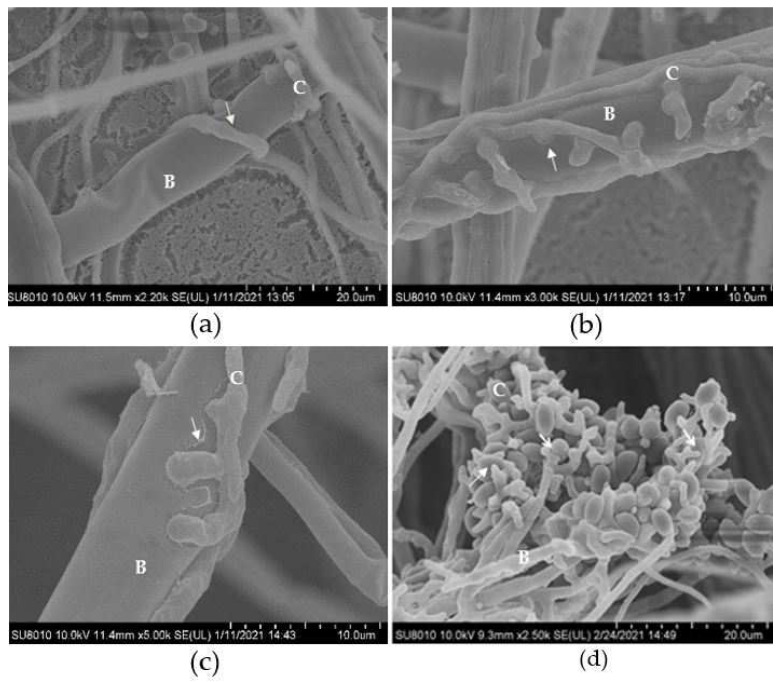
Observation of mycoparasitism of *C. rosea* G67-1 strain to *B. cinerea* under a scanning electron microscope. (**a**) G67-1 hyphae coil around those of *B. cinerea*. (**b**) Attachment to and penetration into *B. cinerea* hyphae by *C. rosea* appressoria. (**c**) Partial degradation of *B. cinerea* cell walls. (**d**) Attachment to and penetration into *B. cinerea* spores. B, *B. cinerea*; C, *C. rosea*.

Under an SEM, it could be clearly seen that the hyphae of *B. cinerea* were tightly wrapped by those of the biocontrol fungus (Figure 6a), and an amount of appressoria generated from the hyphae of *C. rosea*, by which the mycoparasite penetrated the host cells (Figure 6b). At this point, the cell walls of the pathogen were partially degraded (Figure 6c). In addition to attacking fungal mycelia, *C. rosea* could also attack *B. cinerea* spores, and microscopy showed that the newly produced host conidia were penetrated by *C. rosea* appressoria (Figure 6d).

### 3.3. Colonization of C. rosea and Its Mycoparasitic Interaction with B. cinerea on Tomato Leaves

**Figure 7:**
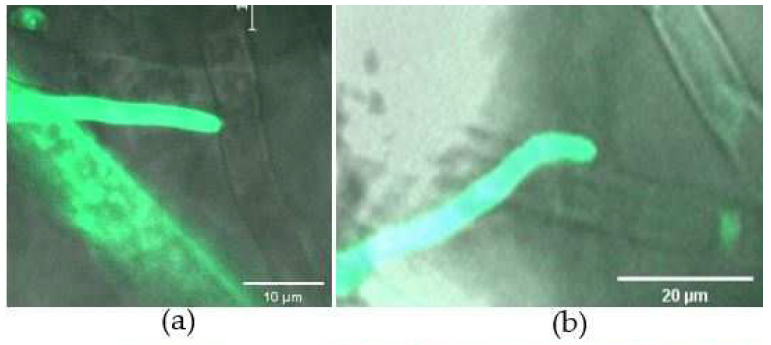

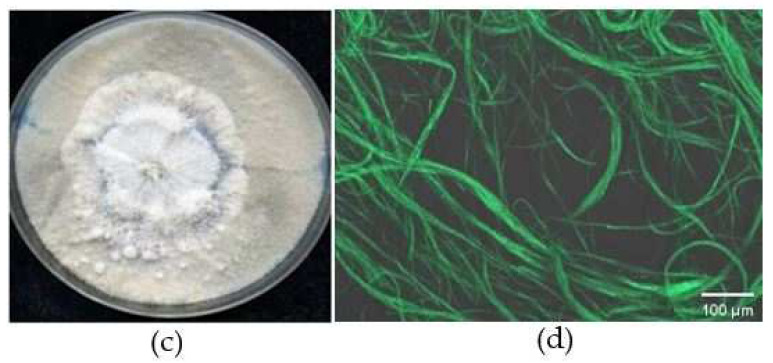
Colonization of *C. rosea* G67-1 strain and its mycoparasitism against *B. cinerea* TC-B1 on tomato leaves observed under a fluorescence microscope. (**a**) G67-1 hyphae growing towards *B. cinerea*. (**b**) G67-1 hyphae growing alongside the mycelia of TC-B1. (**c**) Fungal colonies derived from surface-sterilized tomato leaflets on PDA plates. (**d**) Mycelia of GFP-labelled *C. rosea* and *B. cinerea* isolates overlapping regions of PDA plates.

Three days after inoculation, the hyphae of *B. cinerea* and GFP-labelled *C. rosea* spread on the surface of the leaflets. When encountering the host, the hyphae of the biocontrol fungus grew alongside the mycelia of *B. cinerea*, and developed new branches extending towards the pathogen (Figure 7a,b). *B. cinerea* and *C. rosea* were also isolated from surface-sterilized tomato leaves, and identified by colony morphology and fluorescence microscopy (Figure 7c,d). The results showed that *C. rosea* could colonize on the surface and inside tomato leaves and attack the pathogen.

## 4. Discussion

Using GFP as a marker, interactions between different fungal species can be investigated [4,39,40]. Our current work showed that almost all the GFP transformants of *C. rosea* were stable and fluorescence could be detected in the hyphae and spores of the mutants continuously cultured in plates and in different infection stages, indicating that GFP had been successfully inserted into the genome of the *C. rosea* 67-1 strain. Six mutants with strong fluorescence were assayed, and all GFP-labelled transformants were quite similar to the wild-type strain in terms of hyphal extension, sporulation, and biocontrol activities, ensuring no major biological changes had occurred that might affect the interactions of the mycoparasite and its host.

In a mycoparasitic process, a host is penetrated by a predator and typically utilized as a food source. Understanding the interactions and modes of action of mycoparasites will promote their control effects to plant fungal diseases and facilitate their application in the field. In the present study, the mycoparasitism of *C. rosea* against *B. cinerea* was investigated, during which four stages were identified. Firstly, the biocontrol fungus grew towards its host. A mycoparasite may perceive compounds secreted by a host and initiate chemotactic responses to these signals [41,42]. When confronted with each other, *C. rosea* mycelia extended towards those of *B. cinerea* and produced many papilla-like outgrowths at the tips of the hyphae, where were active developing regions and sensitive to various stimuli. This kind of infection mechanism was also observed in *T. atroviride* when parasitizing *R. solani* and *P. ultimum* [4], and the exudates of *R. solani* could induce the formation of papilla in *T. virens* [43].

Next, the mycoparasite attached to its host. Under a fluorescence microscope, it was noticed that *C. rosea* hyphae attached to those of *B. cinerea* after 3 days of face-to-face cultivation, grew clinging to the pathogen, and stretched many branches to be ready to attack the host. In a previous study of *T. harzianum*, the hyphae were also found to specifically evolve and profusely branch to a powdery mildew species *Oidium longipes* [44]. During this time, the pathogens may secrete glutinous substances that are essential for the parasites to recognize their hosts. It has been evidenced that the interactions of lectin carbohydrate may mediate the recognition and attachment of *Trichoderma* species to soil-borne plant pathogenic fungi [45].

After attachment, *C. rosea* isolates generated specialized structures that penetrate host cells, but such structures are not always present in mycoparasites. No penetration structures were detected in *Sphaerodes quadrangularis*, a facultative biotrophic fungus, when co-cultured with *F. oxysporum* and *F. graminearum* [46]. During the mycoparasitic process, *C. rosea* generated a hook-like structure and appressoria to attack and penetrate the hyphae and spores of *B. cinerea*. Finally, the mycoparasite absorbed nutrients inside the host, and the cells of the pathogen were gradually disintegrated.

It is also essential for biocontrol fungi to secrete chemical compounds to accomplish host invasion. During the interaction, the mycoparasites may produce multiple cell wall degrading enzymes (CWDEs) and secondary metabolites, such as antibiotics and toxins, that accelerate the degradation of the hosts [9,47]. Several enzymes, including chitinase, cellulase and glucanase, were identified in *Trichoderma* isolates when colonizing their hosts [5,48]. In the present study, *B. cinerea* cell walls were damaged when invaded, implying that some hydrolases might be involved in the mycoparasitism of *C. rosea*.

Many mycoparasite species are facultative fungi that can acquire nutrition from various ambient environments, including soil, plant residues, culture media and fungal hosts. Competition for limited nutrients is essential for the survival of microorganisms, and it is considered as one of the important mechanisms for the biological control of plant fungal pathogens [49]. When placed on the opposite sides of slides without any nutrients, the biocontrol fungus attacked its host and developed dedicated infection structures (papilla, coil, hook and appressoria) within 72 h, while on slides containing PDA medium, it was only seen that *C. rosea* hyphae attached and grew parallel to host hyphae after 72 h, suggesting that *C. rosea* might preferentially utilize available nutrients, such as glucose, which delays colonization of the host. Similar results were reported in previous studies, which indicates that the mycoparasites are more likely to form appressoria to take up nutrients if external conditions are poor [50,51] and their biocontrol activities become more effective [52,53].

Biotrophic mycoparasites obtain nutrition from living fungi without killing them. With the penetration structures or absorptive cells, the parasites build close relationships with their hosts, maintaining their survival and propagation [54]. However, for necrotrophic mycoparasites, when encountering potential preys, they may produce toxic compounds and degrading enzymes to kill the hosts and facilitate their invasion. Once colonization is established, the parasites take up nutrients from the dead cells and proliferate in the hosts. In general, most mycoparasitic species are necrotrophs, but they may have short biotrophic phases [55,56,57]. In our current study, it was seen that the *C. rosea* hyphae grew close to those of the host, produced appressorium-like structures by which they penetrated the mycelia and spores of *B. cinerea*, and decomposed the hyphae of the pathogen when the necrotrophic phase was initiated. In the area in which *T. atroviride* and *R. solani* interacted, the swelling of the hyphae of the biocontrol fungus was observed [4]. Further, when some *Trichoderma* species attach to prey, it is often followed by coiling around host hyphae and forming appressorium-like structures that assist penetration [58]. However, obligate biotrophic mycoparasites, such as *Verticillium biguttatum*, *Gliocephalis hyaline* and *Olpitrichum tenellum*, can only survive on living organisms and cannot grow on culture media [54,59,60]. Our results show that *C. rosea* can produce penetration structures and propagate in pathogenic fungi, and can also colonize on plants and within plant tissues, suggesting that *C. rosea* is a promising facultative mycoparasite with great advantages for targeting plant fungal diseases whenever pathogens invade.

## 5. Conclusions

In this study, a highly efficient *C. rosea* 67-1 strain was tagged with GFP, and its interaction with *B. cinerea* was investigated by fluorescence confocal microscopy and SEM. When encountering the host, *C. rosea* produces dedicated infection structures, such as papilla, coil, and appressoria, to attack and penetrate the pathogen, absorbs nutrients and disintegrates the pathogen cells. *C. rosea* can also colonize on tomato leaves and thereby, protect the plants from infection. To the best of our knowledge, this is the first report of infection structures formed in *C. rosea* during the mycoparasitic process. The findings are of great significance to reveal the mechanisms of the biocontrol fungus and promote the development and application of *C. rosea* biocontrol agents.

## Figures and Tables

**Table 1:** Control efficacy of GFP-labelled *C. rosea* 67-1 transformants against soybean Sclerotinia stem rot.

Strain	Disease Index	Control Efficacy (%)
G67-1	28.4 ± 1.7 b	55.9 ± 1.9 a
67-1	26.9 ± 1.9 b	58.2 ± 2.2 a
CK	64.3 ± 1.1 a	-

Data are means ± standard deviation (SD) of three replicates. Different letters in a column indicate significant differences at *p* < 0.05.
